# Detection and Localization of Prostate Cancer With the Targeted Biopsy Strategy Based on ADC Map: A Prospective Large-Scale Cohort Study

**DOI:** 10.1002/jmri.23587

**Published:** 2012-01-13

**Authors:** Yuji Watanabe, Akito Terai, Tohru Araki, Masako Nagayama, Akira Okumura, Yoshiki Amoh, Takayoshi Ishimori, Mana Ishibashi, Satoru Nakashita, Yoshihiro Dodo

**Affiliations:** 1 Department of Radiology, Kurashiki Central HospitalKurashiki, Japan; 2 Department of Urology, Kurashiki Central HospitalKurashiki, Japan; 3 Araki Urologic ClinicKurashiki, Japan

**Keywords:** magnetic resonance imaging, prostate cancer, targeted biopsy, transition zone, diffusion-weighted imaging, apparent diffusion coefficient

## Abstract

**Purpose:**

To investigate the usefulness of targeted biopsy strategy based on apparent diffusion coefficient (ADC) maps in the detection and localization of prostate cancer.

**Materials and Methods:**

Institutional review board approval and informed consent from all participants were obtained. This study included 1448 consecutive patients suspected of having prostate cancer based on PSA level, who were divided into two groups: Group A included 890 patients with low-ADC lesions who underwent targeted and systematic biopsies; Group B included 558 patients with no low-ADC lesions who underwent only systematic biopsies. The cancer detection rates (CDR) of each group, positive predictive value (PPV), and negative predictive value (NPV) of ADC maps were calculated.

**Results:**

The CDR was 70.1% for Group A, higher than those for overall patients (48.1%) and for Group B (13.1%) with significant difference (*P* < 0.001). In the serum, PSA range from 4 to 20 ng/mL, the CDR was higher for the Group A than for the Group B and overall patients with significant differences. PPV and NPV of MR findings were 70.1% and 86.9%, respectively. Especially, the PPV of the MR findings for the anterior portion was as high as 90.1%. Among the false negatives of MR findings, Gleason score proved 6 or smaller in 79.5%, and positive core number was merely one or two in 80.8%.

**Conclusion:**

The targeted biopsy strategy based on ADC maps can be useful in the detection and localization of prostate cancer with high PPV. J. Magn. Reson. Imaging 2012;35:1414–1421. © 2012 Wiley Periodicals, Inc.

The Ideal Biopsy protocol to perform to detect all, or at least the majority of, prostate cancers has been a controversial topic, and subject to considerable change in the modern biopsy era. Ultrasound-guided prostate biopsies to take six cores were first recommended. Since the late 1990s, this recommendation was changed to 10–12 cores, on the basis of data showing that the more-extensive biopsies resulted in the detection of 30 % more cancers than the conventional sextant biopsy (1, 2). Saturation biopsies might be advised in patients with repeated negative results from standard biopsies and for whom persistent suspicion of prostate cancer exists on the basis of PSA level (1).

Correlation studies comparing biopsy results to those obtained from radical prostatectomy specimens (3, 4) or autopsied material (5, 6) have raised main issues that cancers are frequently missed on initial biopsy and are mischaracterized in terms of size, location and grade. Anterior portion seems to be very special in the detection of cancer, because anterior portion cancers could be missed by standard peripheral zone biopsies (7) and require additional sets of biopsies before detection (8). Together with the aid of novel imaging technique, suspicious foci could be sampled through targeted prostate biopsies, which could increase cancer detection rate and improve characterization.

MR imaging of the prostate gland with endorectal coil or pelvic phased array coil has been widely used to detect and localize malignant lesions which mainly occurs in the peripheral zone (9, 10). Although some studies demonstrated the added value of T2-weighted MR imaging and MR spectroscopy in localizing prostate cancer compared with endorectal ultrasonography (10, 11), MR imaging has been generally considered inadequate for use in the evaluation of transition zone cancers because of heterogeneous T2 signal intensity in the normal transition zone(12). Recently, several investigators have reported the potential usefulness of diffusion-weighted imaging (DWI) and apparent diffusion coefficient (ADC) map for detecting prostate cancer, which shows lower ADC than a normal peripheral zone and a nonmalignant transition zone (13–15).

In this prospective large-scale cohort study, we aimed to present the usefulness of targeted biopsy strategy based on ADC maps in the detection and localization of prostate cancer.

## MATERIALS AND METHODS

### Patient Population

The prospective large-scale cohort study enrolled 1448 consecutive patients (mean age and SD: 72 ± 7.5 years old, range from 50 to 89) suspected of having prostate cancer because of either rising or elevated PSA level (4.0 ng/mL or higher) between October 2004 and October 2008. This study was approved by the institutional review board. Before being enrolled in this study, each patient gave written informed consent. All the patients underwent MR examination before prostatic biopsies and were divided into two groups according to the MR findings: Group A included the patients who had low-ADC lesions and underwent targeted biopsies of the suspicious foci in addition to systematic biopsies, and Group B included the patients with no suspicious low-ADC lesions who underwent only systematic biopsies.

### MR Imaging

MR imaging was performed on a 1.5 Tesla super-conductive magnet system (Gyroscan Achieva; Philips Medical systems, Best, the Netherlands). Each patient was positioned supine on the table with the synergy cardiac coil wrapped around the pelvis. After initial T1-weighted localizing images were obtained, MR images of the entire prostate gland and seminal vesicle in the transaxial direction were acquired with DWI and T2-weighted turbo spin echo (TSE) imaging. The scan parameters are listed in Table 1.

**Table 1:** Scan Parameters of the Pulse Sequence Used for the Prostatic MR Imaging

MR imaging	Diffusion-weighted imaging	T2-weighted imaging
Pulse sequence	Spin-echo echo-planar-imaging	3D T2-weighted TSE
Direction	Transaxial	Transaxial
TR/TE (ms)	6300/50	1500/150
Section thickness (mm)	2.5	1.4
Overlap (mm)	0	0.7
Field of view (mm)	350	200
Matrix	144 × 256 (interpolated to 256 × 256)	224 × 512 (interpolated to 512 × 512)
Voxel size (mm^3^ )	1.37 × 1.37 × 2.5	0.4 × 0.4 × 0.7
Signal acquisition	7	2
Scan time	5 min 21sec	7 min 27 sec
Multiplanar reconstruction thickness (mm)	4	4
Reconstruction direction	Transaxial, coronal, sagittal	Transaxial, coronal, sagittal
Others	b-value s/mm^2^ of 0 and 600, ADC map reconstruction: 2.5 mm-thick TRS, 4 mm-thick TRS, COR, SAG	Echo train length of 61

DWI was performed with spin-echo echo-planar-imaging sequence in the straight transaxial direction. The scan parameters were b values of 0 and 600 s/mm^2^, TR/TE 6300/50, section thickness of 2.5 mm without intersection gap, field of view of 350 mm, matrix of 144 × 256 that was interpolated to 256 × 256, voxel size of 1.37 × 1.37 × 2.5 mm^3^, seven signal acquisition, and scan time of 5 min 21 s. The ADC maps with 2.5-mm-thick sections without intersection overlap were constructed from ADC values calculated from signal intensity data obtained in the diffusion weighted images with b values 0 and 600 s/mm^2^. Then, by using the 2.5-mm-thick ADC maps, the transaxial, coronal and sagittal ADC-maps were reconstructed with 4-mm-thick gapless sections to match 4-mm-thick sections of the following T2-weighted images.

For T2-weighted imaging, thin-section high-spatial-resolution three-dimensional T2-weighted TSE images were obtained in the straight transaxial plane with the following parameters: TR/TE 1500/150 ms; echo train length of 61, section thickness of 1.4 mm with intersection overlap of 0.7 mm; field of view of 200 mm; matrix of 224 × 512 that was interpolated to 512 × 512; reconstructed voxel size of 0.4 × 0.4 × 0.7 mm^3^; and number of excitation of two. Total acquisition time was 7 min 27 s. Then, transaxial, coronal and sagittal images were reconstructed with 4-mm-thick gapless sections, which provided anatomical details of the prostate gland.

#### Image Analysis

All the images were analyzed by the two radiologists (YW, MN) with consensus, who were aware of patients' serum PSA level, with use of soft-copy reading at an electronic workstation (Shade-Quest; Yokogawa Electronic Co, Tokyo, Japan). The readers first evaluated ADC maps on the display in the standard gray scale mode with window width and level set at 2400 and 1350 to make areas of ADC value of 1.35 × 10^−3^ mm^2^ /s appear as median gray color, which facilitates to visually detect low-ADC lesions of 5mm or greater with ADC value of 1.35 × 10^−3^ mm^2^ /s or less. ADC values of the low-ADC lesions were measured on 2.5-mm-thick transaxial sections with elliptical or multiangular user-defined regions-of-interest (ROI) drawn over the low-ADC lesions. The region of interest was as large as possible to cover the lesion and minimize noise. The diameter of the region of interest was approximately 70–80% of the diameter of the lesion. Then, the short and long diameter of the lesion was measured on the corresponding T2-weighted image which was matched with the ADC map by using slice-location function.

The criteria to select lesions for targeted biopsy were the following MR imaging features: low-ADC lesions of the short diameter of 5mm or greater with ADC value of 1.35 × 10^−3^ mm^2^ /s or less. Then, exclusion criteria to eliminate benign lesions were applied for the selected lesions, by using the characteristics indicative of hyperplastic nodules on T2-weighted images: inhomogeneous signal intensity with high-intensity spots, round shape, and smooth margin with hypointense pseudocapsule (12, 16). Special cautions were given not to mistake normal central zone for low-ADC lesion to be targeted at biopsy (17), because a normal central zone mimics a malignant lesion by its low ADC value. T2-weighted images in both coronal and transaxial planes were used for identifying normal central zone with the following typical findings: symmetric anatomical location overlying transition zone and crescent shape surrounding ejaculatory duct with homogeneous T2-low signal intensity.

Then, the low-ADC lesions finally selected for the subsequent targeted biopsy were recorded with regard to location, shape, and size (short and long diameter). To report location of a low-ADC lesion, transition and peripheral zones were divided into the upper, middle and lower third segments: the upper third segment which included the region in the base of prostate, the middle third segment which included the region at the level of the verumontanum, and the lower third segment which included the remaining inferior portion. The left and right sides were separated by the median sagittal plane through the verumontanum. Direction in the transverse plane was also described as a time of the clock. Special attention was paid to the “anterior portion” indicating the portion anterior to the prostatic urethra including transition and/or peripheral zone in the direction from 10 to 2 o'clock, in which cancer foci would be missed only at systematic biopsies.

#### Biopsy Protocol

According to the MR results, patients were divided into the Group A and B. In the Group A, all the patients underwent 10–12 core biopsies including 8 core systematic biopsies and 2–4 core targeted biopsies from the suspicious foci detected on ADC maps. In the Group B, all the patients, who had no suspicious foci detected on ADC maps, underwent 8 core systematic biopsies.

**Figure 1:**
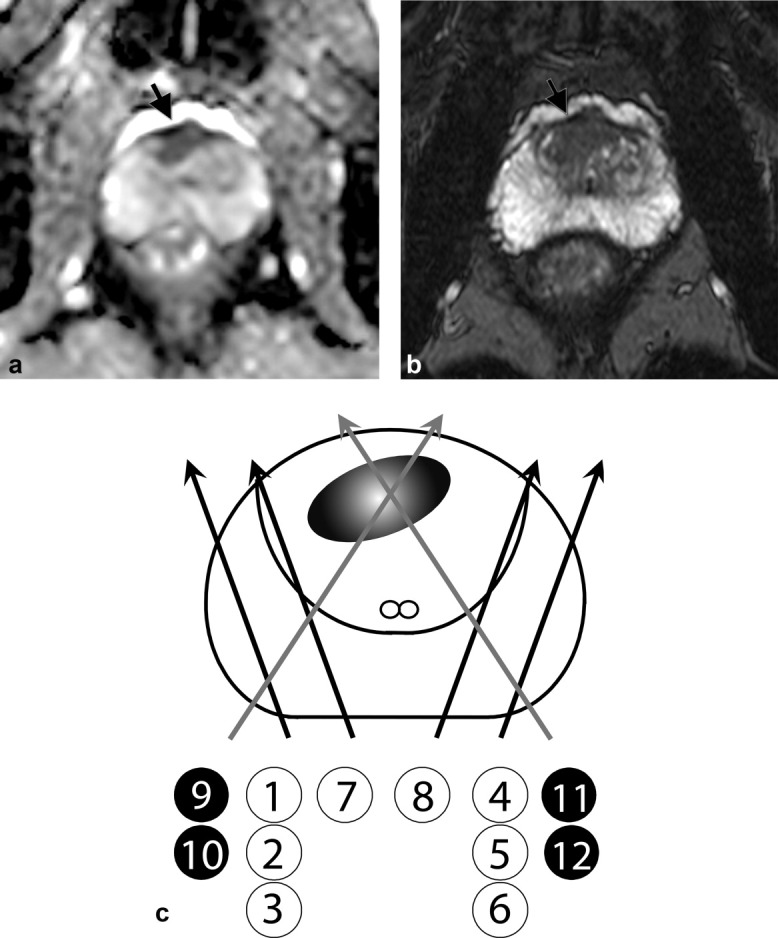
Prostate cancer in the anterior middle transition zone and fibromuscular stroma in a 72-year-old patient with high serum prostate-specific antigen (PSA) level of 13.0 ng/mL. **a:** Apparent diffusion coefficient (ADC) map. **b:** T2-weighted image. **c:** Scheme of systematic and targeted biopsies. ADC map (a) demonstrates a crescent-shaped low-ADC (0.96 × 10^−3^ mm^2^ /s) lesion (arrow) in the anterior middle transition zone and fibromuscular stroma of the prostate gland. The lesion (arrow) shows low signal intensity on T2-weighted image (b). The scheme of prostate biopsy (c) demonstrates the number, location and course of all the biopsy specimens including eight systematic biopsy cores (black arrows) and four targeted biopsy cores (gray arrows). Histological examination reveals well-differentiated adenocarcinoma only in the targeted biopsy specimens.

**Figure 2:**
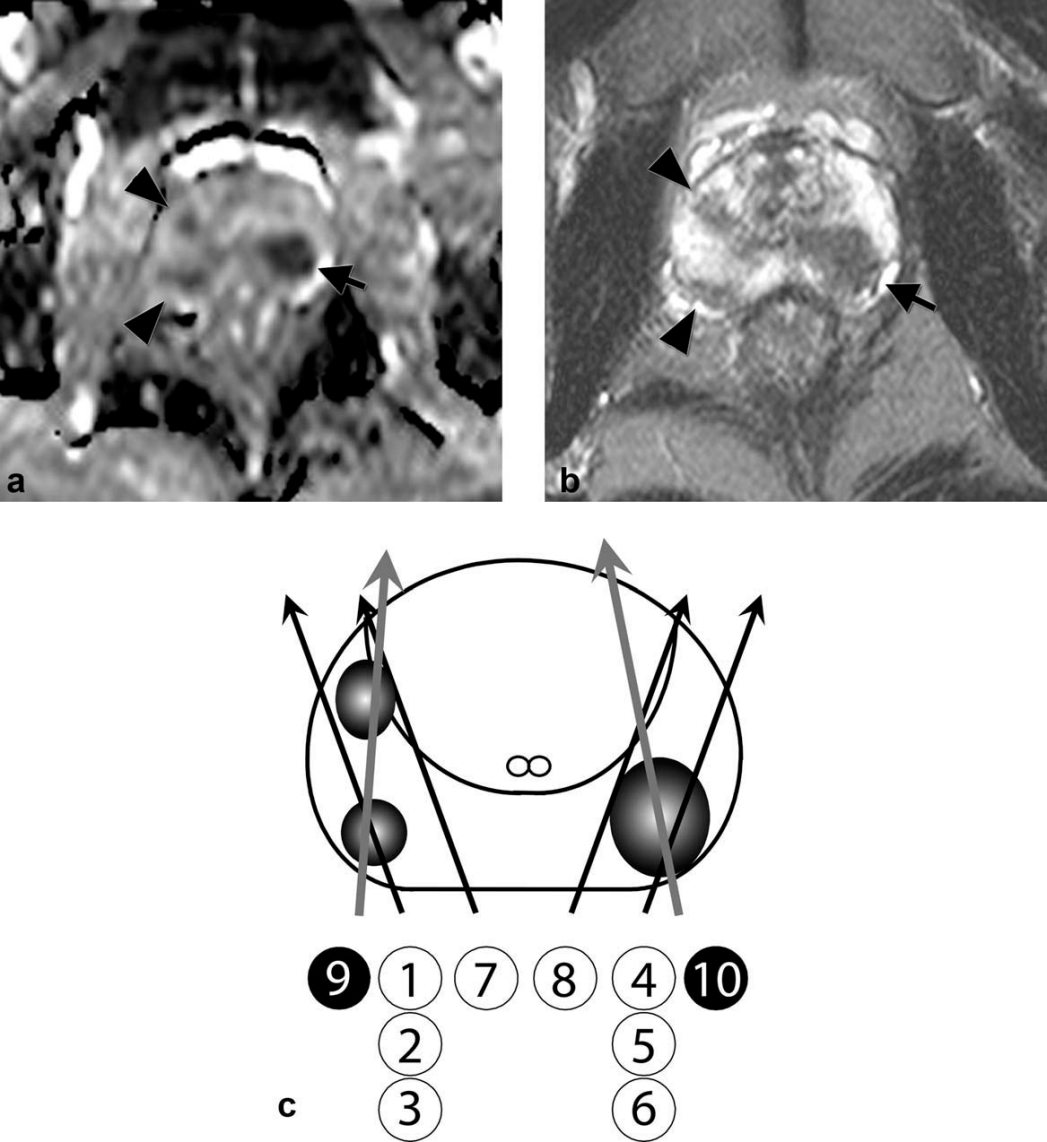
Multiple prostate cancer foci in the peripheral zone in a 60-year-old patient with high serum PSA level of 22.98 ng/mL. **a:** Apparent diffusion coefficient (ADC) map. **b:** T2-weighted image. **c:** Scheme of systematic and targeted biopsies. ADC map (a) demonstrates an oval low-ADC (0.77 × 10^−3^ mm^2^ /s) lesion (arrow) in the left middle peripheral zone and two small low-ADC (1.09 × 10^−3^ mm^2^ /s) lesions (arrowheads) in the right middle peripheral zone. All the lesions (arrow, arrowheads) show low signal intensity on T2-weighted image (b). The scheme of prostate biopsy (c) demonstrates the number, location and course of all the biopsy specimens including eight systematic biopsy cores (black arrows) and two targeted biopsy cores (gray arrows). Histological examination proves both the lesions to be moderately-differentiated adenocarcinoma in both the targeted and systematic biopsy specimens.

**Figure 3:**
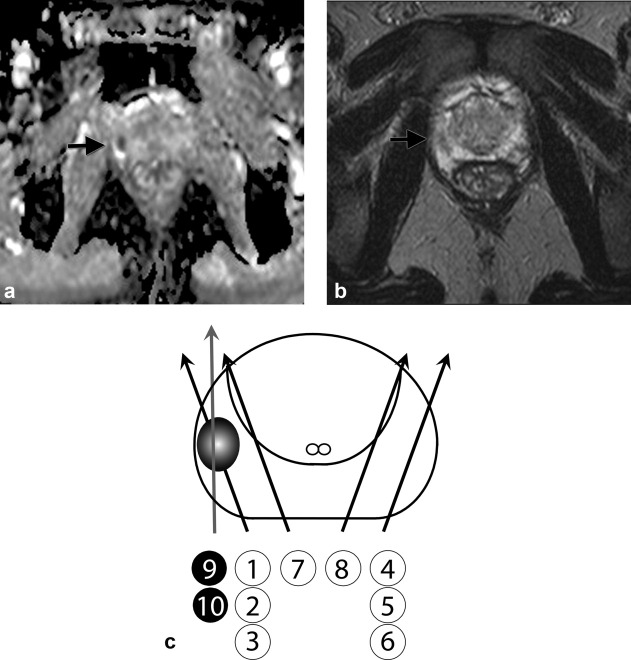
Prostate cancer in the right middle peripheral zones in a 59-year-old patient with high serum PSA level of 10.5 ng/mL. **a:** Apparent diffusion coefficient (ADC) map. **b:** T2-weighted image. **c:** Scheme of systematic and targeted biopsies. ADC map (a) demonstrates a crescent-shaped low-ADC (1.09 × 10^−3^ mm^2^ /s) lesion (arrow) in the right middle peripheral zone. The lesion (arrow) shows low signal intensity on T2-weighted image (b). The scheme of prostate biopsy (c) demonstrates the number, location and course of all the biopsy specimens including eight systematic biopsy cores (black arrows) and two targeted biopsy cores (gray arrows). Histological examination proves the peripheral zone lesion to be moderately-differentiated adenocarcinoma in both the targeted and systematic biopsy specimens.

Biopsies were performed by the urologists (at, ta, and two other urologists working with them) and taken with a spring-loaded Pro-Mag Ultra automatic biopsy instrument (MD TEC, Denmark) and an 18-gauge needle under guidance of transrectal ultrasonography (TRUS). Eight-core systematic biopsies were performed with six lateral cores that were directed to the lateral peripheral zones at the base, middle and apex and two paramedian cores that were directed to the mid parasagittal peripheral and transition zones at the middle gland. The 2–4 targeted biopsy cores were taken from the segments of the suspicious foci corresponding to the selected low-ADC lesions according to the MR reports (Figs. 1–3). In each patient, maximum four largest low-ADC lesions were sampled through the targeted biopsy cores. When a patient had a single low ADC lesion to be targeted at biopsy, two biopsy cores were directed toward the single targeted lesion. When a patient had two-to-four low ADC lesions to be targeted, a single biopsy core was directed toward each of the targeted lesions.

Targeted biopsies were performed under US guidance as follows; when a suspicious focus corresponding to the low ADC lesion was detected as a low-echoic lesion on TRUS, targeted biopsy of the suspicious focus was readily performed under US guidance. When a targeted lesion was not detected on TRUS, targeted biopsy cores were directed to the suspicious focus according to the MR report describing location, size and direction.

In each patient, all the biopsy-core specimens obtained were numbered. Those locations and biopsy courses were recorded on a scheme of prostate gland so that each core was individually identified. Each core specimen was marked at the rectal-side tip with black ink, and examined histologically.

#### Histological Evaluation of Biopsy Specimens

For each patient, biopsy-core specimens served as the reference standard. Histological slides of the specimens were examined and read by the three pathologists who were given the information of the MR results and biopsy schemes. Histopathological diagnosis was done by consensus reading. Histological results were reported by the pathologists about presence or absence of prostate cancer. When prostate cancer was histologically diagnosed, the following details of the biopsy cores were also reported; the sample number of positive result, the location of the cancer on the core, distance from the rectal-side tip, a proportion in length of the cancer occupying in the positive sample core, and differentiation type of cancer. Gleason score of the cancer was also reported.

### Statistical Analysis

Histopathology was the standard of reference, and all histopathology results were included in the case record form. The cancer detection rates (CDR) of the Group A, B, and overall patients were calculated according to the PSA ranges. The positive predictive value (PPV) and negative predictive value (NPV) of the MR findings in the detection of prostate cancer were also calculated. The PPV of the MR results for the anterior portion was also calculated. In the Group B, positive systematic biopsy results that serve as false negatives of the MR findings were characterized according to the Gleason scores and PSA ranges.

Chi-square test was used to compare CDRs among the Group A, B, and overall patients. In every statistical analysis, significance was considered to exist when *P* value was less than 0.05.

## RESULTS

Among the 1448 patients overall, the Group A included 890 patients who underwent both systematic and targeted biopsies of suspicious foci detected on ADC maps, and the Group B included 558 patients who had no suspicious foci detected on ADC maps and underwent only systematic biopsies (Table 2).

**Table 2:** Cancer Detection Rates for Group A, B and Overall Patients According to the Range of PSA Levels

	Overall patients			Group A			Group B			*P* value	
				
serum PSA level ng/mL	No.	%	95% CI	No.	%	95% CI	No.	%	95% CI	Group A vs Group B	Group A vs total
PSA < 4	20/77	26.0	16–36	17/41	41.5	26–57	3/36	8.3	0–17	<0.001	0.0842
4 <= PSA <= 10	385/928	41.5	38–45	331/501	66.1	62–70	54/427	12.6	9–16	<0.001	<0.001
10 < PSA <= 20	145/264	54.9	49–61	133/185	71.9	65–78	12/79	15.2	7–23	<0.001	<0.001
PSA > 20	147/179	82.1	77–88	143/163	87.7	83–93	4/16	25.0	4–46	<0.001	0.1492
Total	697/1448	48.1	46–51	624/890	70.1	67–73	73/558	13.1	10–16	<0.001	<0.001

The CDR was 70.1% (95%CI: 67–73) for the Group A and higher than those for the overall patients (48.1%; 95%CI: 46–51) and for the Group B (13.1%; 95%CI: 10–16). There were significant differences (*P* < 0.001) in the CDRs among them. In the serum, PSA range from 4 to 20 ng/mL, the CDR was higher for the Group A than for the Group B, and overall patients with significant differences, whereas there was no significant difference between the overall patients and the Group A in the serum PSA levels lower than 4 ng/mL and higher than 20 ng/mL (Table 2). In the Group A, the CDR elevated as the PSA ranges increased.

The PPV and NPV of the MR findings for the detection of prostate cancer were 70.1% (95%CI: 67–73) and 86.9% (95%CI: 84–90), respectively (Table 3) (Figs. 1–3). Especially, the PPV of the MR findings for the anterior portion was 90.1% (95%CI: 85–95) in the 127 of 141 patients (Table 4) (Fig. 1).

**Table 3:** Positive Predictive Value and Negative Predictive Value of the MR Findings for the Detection of Prostate Cancer

	MR findings for the cancer detection		
	
	No.	%	95% CI
PPV	624/890	70.1	67–73
NPV	485/558	86.9	84–90

**Table 4:** Positive Predictive Value of the MR Findings for the Anterior Portion

	Positive predictive value		
	
	No.	%	95% CI
Anterior portion	127/141	90.1	85–95

In the Group B, 73 (13.1 %) out of the 558 patients had prostate cancer. Among the 73 patients, Gleason score proved to be 6 or smaller in 58 (79.5%), 7 in 6 (8.2%) and 8 or greater in 3 (4.1%). Positive core number was one or two in 59 (80.8 %) and three or more in 14 (19.2 %) (Table 5).

**Table 5:** Characteristics of the Positive Systematic Biopsy Results in the Group B

	No.	%	95% CI
Gleason score			
6 or smaller	58/73	79.5	70–89
7	6/73	8.2	2–15
8 or greater	3/73	4.1	0–9
not defined	6/73	8.2	2–15
Positive core number			
n=1	42/73	57.5	46–69
n=2	17/73	23.3	14–33
n=3	7/73	9.6	3–16
n=4 or more	7/73	9.6	3–16

## DISCUSSION

The ideal biopsy protocol and number of biopsies to perform to detect all or at least the majority of prostate cancers has been a controversial topic. Cancer detection rate varies depending on biopsy protocols. Standard peripheral zone biopsies were previously reported to yield a CDR of 30–35% (1, 18). Other authors reported the initial saturation biopsy and 10–12 core conventional extended biopsy yielded a CDR of 44.6% and 51.7%, respectively (1, 19). Although higher number of cores such as saturation biopsies may be believed to help to identify more prostate cancers, it was reported that the CDR of the 21-core biopsy strategy was similar to that of their 10-12-core biopsy scheme as an initial biopsy regimen (20).

The overall CDR of 48.1% obtained in this study seems to be comparable to those reported previously by using the initial saturation biopsy and 10-12-core conventional extended biopsy (1, 19) and to be higher than that reported by using standard peripheral zone biopsies (18). In addition, the 10-12-core targeted biopsy strategy performed in the Group A, consisting of 8 systematic biopsy cores and 2-to-4 additional targeted biopsy cores, yielded much higher CDR of 70.1% in comparison to that of 13.1% obtained by the eight-core systematic biopsy performed in the Group B. These results seem to have been acquired not only by higher number of biopsy cores but also by both the patient selection with ADC maps and the targeted biopsies of the suspicious foci detected on ADC maps. This finding may suggest that MR imaging with ADC maps can be very useful in the selection of patients for subsequent prostate biopsy and in the detection of biopsy targets, which could increase CDR by the incorporation of the targeted biopsy strategy.

The cutoff level of ADC could play an important role as an objective index in the detection and localization of suspicious foci of prostate cancer with ADC maps. The cutoff level should be determined in way to achieve the best sensitivity, specificity, and accuracy in the diagnosis of prostate cancer (21). Recent studies have proposed the cutoff level of 1.4 to 1.6 × 10^−3^ mm^2^ /s for the diagnosis of the peripheral zone cancer with high sensitivity and specificity (22). Benign hyperplastic nodules, however, may show low ADC and give false positive results (17). Therefore, a lower cutoff level would need to be applied to increase specificity in the detection of transition zone cancer. The 1.35 × 10^−3^ mm^2^ /s used in this study, which was determined by using our data obtained from the 45 radical prostatectomy specimens, seems to be very reasonable in the detection of suspicious foci of prostate cancer (21). Furthermore, thin-slice technique with 2.5-mm-section thickness used for diffusion-weighted imaging in this study might minimize influence of the partial volume averaging on ADC measurement and provide relatively accurate information about ADC value of suspicious foci.

There is a major limitation of this study with regard to matching the MR reports with targeted TRUS biopsies. It should be important to accurately take a sample from the suspicious foci detected on MR imaging. The targeted biopsy protocol used in this study may lack in precise consistency with the MR and TRUS biopsy results. Ultrasound-guided biopsy under real-time ultrasonography-MRI fusion-guidance might give precision in taking a sample from the suspicious lesion corresponding to the MR reports. However, a special device and fusion soft-ware for US and MR images should be necessary (23). More targeted biopsy cores focusing on a single suspicious lesion could be another remedy for this problem. In this study, the Group A demonstrated very high CDR of 70.1% by the selection of patients with ADC results and by adding 2–4 targeted biopsy cores for the suspicious foci detected on ADC maps, in comparison with the CDR of 13.1% I the Group B. Especially, anterior portion cancers, which might have been missed only with the standard systematic biopsies (7, 8), were detected and identified with very high positive predictive value of 90.1%, These results will suggest that the targeted biopsy strategy based on ADC map can detect more cancer foci than the standard systematic biopsies.

Another limitation will be cancer foci missed by MR imaging in both the Group A and B of this study. In the Group B which works as a control for the Group A, 13.1% were found to have prostate cancer and to be false negatives of MR imaging. The majority could be so-called “insignificant cancers” characterized histologically as being low volume (< 0.5 cm^3^ ) and low grade (Gleason score < 7) (1, 24, 25), and might not need radical or potentially harmful treatment (26). Of the false negatives, however, 4.1% was found to have aggressive cancer with Gleason score 8 or greater and 19.2% showed three or more positive biopsy cores. Despite the low prevalence of aggressive or extensive cancer missed by MR imaging, close follow-up examination of PSA should be necessary in patients with negative MR results if MR imaging with ADC maps can be used for the screening of patients for subsequent prostate biopsies.

In summary, this prospective large-scale cohort study demonstrated that the targeted biopsy strategy based on ADC maps can be useful in the patient selection for subsequent prostate biopsies and in the detection and localization of prostate cancer with high accuracy.
